# Cinnamicaldehyde regulates the expression of tight junction proteins and amino acid transporters in intestinal porcine epithelial cells

**DOI:** 10.1186/s40104-017-0186-0

**Published:** 2017-08-16

**Authors:** Kaiji Sun, Yan Lei, Renjie Wang, Zhenlong Wu, Guoyao Wu

**Affiliations:** 1 State Key Laboratory of Animal Nutrition, College of Animal Science and Technology, China Agricultural University, Beijing, 100193 China; 2 DadHank (Chengdu) Biotech Corp, Sichuan, China; 3 Department of Animal Science, Texas A&M University, College Station, TX 77843 USA; 4 Department of Animal Nutrition and Feed Science, China Agricultural University, Beijing, 100193 China

**Keywords:** Amino acid transporters, Barrier function, Cinnamicaldehyde, Intestinal epithelial cells, Tight junction proteins

## Abstract

**Background:**

Cinnamicaldehyde (CA) is a key flavor compound in cinnamon essential oil possessing various bioactivities. Tight junction (TJ) proteins are vital for the maintenance of intestinal epithelial barrier function, transport, absorption and utilization of dietary amino acids and other nutrients. In this study, we tested the hypothesis that CA may regulate the expression of TJ proteins and amino acid transporters in intestinal porcine epithelial cells (IPEC-1) isolated from neonatal pigs.

**Results:**

Compared with the control, cells incubated with 25 μmol/L CA had increased transepithelial electrical resistance (TEER) and decreased paracellular intestinal permeability. The beneficial effect of CA on mucosal barrier function was associated with enhanced protein abundance for claudin-4, zonula occludens (ZO)-1, ZO-2, and ZO-3. Immunofluorescence staining showed that 25 μmol/L CA promoted the localization of claudin-1 and claudin-3 to the plasma membrane without affecting the localization of other TJ proteins, including claudin-4, occludin, ZO-1, ZO-2, and ZO-3, compared with the control cells. Moreover, protein abundances for rBAT, xCT and LAT2 in IPEC-1 cells were enhanced by 25 μmol/L CA, while that for EAAT3 was not affected.

**Conclusions:**

CA improves  intestinal mucosal barrier function by regulating the distribution of claudin-1 and claudin-3 in enterocytes, as well as enhancing protein abundance for amino acid transporters rBAT, xCT and LAT2 in enterocytes. Supplementation with CA may provide an effective nutritional strategy to improve intestinal integrity and amino acid transport and absorption in piglets.

## Background

Cinnamicaldehyde (CA) is a key flavor compound in cinnamon essential oil extracted from the stem bark of *Cinnamomum cassia* in nature [1]. CA is widely used in the perfume, pharmacy, and food processing industries due to its antioxidant, anti-microbial, and anti-diabetic properties [2–6]. Moreover, it has been reported that CA has chemotherapeutic and anticancer effects through the inhibition of proliferation, inducing apoptosis, and blocking angiogenesis [7–9]. Importantly, CA is a safe flavor compound approved by FDA and the ‘Flavor and Extract Manufacturers’ Association of the United States, suggesting the potential administration of this dietary factor may be achievable within an acceptable safety range  for humans and animals [5]. The well documented antimicrobial properties and safety of CA have promoted its application to the nutrition of  humans and animals. It has been reported that supplementation of essential oils, in which CA is the major component, increases the digestibility of crude protein in weaned pigs [10]. The beneficial effects of CA are associated with the enhanced secretion of digestive enzymes, improved nutrient digestion, and enhanced feed intake [11–14]. It remains unknown whether CA has any effect on intestinal barrier function, as well as nutrient transport and absorption in humans and pigs.

The epithelial barrier is formed by the apical plasma membrane and intercellular tight junction (TJ), which provides physical and functional barriers to prevent bacteria, endotoxins and other harmful substances from entering the blood circulation, while also allowing for the absorption of nutrients [15]. Diverse physiological or pathological stimuli can regulate the intestinal mucosal-barrier function, which contributes to nutrient transport, absorption, and intracellular homeostasis [16–19]. Consistently, dysfunction of TJ proteins has been reported to be associated with increased paracellular permeability, and the development and progression of multiple intestinal disorders [20].

Although CA is known to improve the digestibility of dietary fiber, lipids, and crude protein, the underlying cellular and molecular mechanisms remain largely unknown. We have hypothesized that CA may up-regulate the expression of TJ proteins and amino acid transporters in intestinal epithelial cells, thus contributing to the intestinal barrier function in neonates. This hypothesis was tested in the present study using porcine intestinal epithelial cells (IPEC-1), isolated from neonatal pigs.

## Methods

### Reagents

Dulbecco’s modified Eagle’s F12 Ham medium (DMEM-F12) and fetal bovine serum (FBS) were purchased from Invitrogen (Carlsbad, CA, USA). Epidermal growth factor was a product of BD Biosciences (Carlsbad, CA, USA). Trypsin/EDTA was procured from Gibco (Carlsbad, CA, USA). Antibodies against occludin, claudin-1, claudin-3, claudin-4, zonula occludens (ZO)-1, ZO-2, and ZO-3 were products of Invitrogen (Carlsbad, CA, USA). Unless indicated, all other chemicals including CA were purchased from Sigma-Aldrich (St. Louis, MO, USA).

### Cell culture

Intestinal porcine epithelial cell line 1 (IPEC-1) cells, which were isolated from the jejunum of newborn pigs without access to milk or any food [21], were then cultured in a DMEM-F12 medium supplemented with 5% FBS, insulin (5 μg/mL), transferrin (5 μg/mL), selenium (5 ng/mL), epidermal growth factor (5 μg/L), penicillin (50 μg/mL) and streptomycin (4 μg/mL) as previously described [22]. All cell cultures were carried out at 37 °C in a humidified incubator containing 5% CO_2_.

### Measurement of transepithelial electrical resistance (TEER)

The tightness of the TJ was assessed by measuring TEER as previously described [23]. Briefly, IPEC-1 Cells (5 × 10^4^ cells per well) were seeded in culture transwells (the membrane area, 0.33 cm^2^; pore size, 0.4 μm) which were placed in 24-well culture plates. Cells were incubated with 0, 12.5, or 25 μmol/L CA for the indicated time periods. TEER was determined every 12 h by using a Millicell ERS-2 Voltage-Ohm Meter (World Precision Instruments) equipped with a STX01 electrode as described here [24]. All values are expressed as percentages of the basal level for the controls.

### Monolayer paracellular permeability determination

Paracellular permeability was determined as previously described [25]. Briefly, IPEC-1 cells were seeded in culture transwells as for TEER determination. 1 mg/mL FITC-dextran (20 kDa) was added to the apical side of the monolayer and the flux of FITC-dextran was determined by serially sampling the basolateral compartment every 12 h. The concentration of FITC-dextran was measured using the SpectraMax M3 Multi-Mode Microplate Reader (Molecular Devices) with excitation and emission wavelengths of 490 and 520 nm, respectively. The permeability of monolayer cells was defined as the amount of FITC-dextran that was transported from the apical side into the basolateral chamber. FITC-dextran concentration was calculated by subtracting the fluorescence value of the FITC-free medium.

### Western blot analysis

IPEC-1 cells treated with various concentrations of CA for 24 h were harvested for the analysis of the abundance of TJ proteins, as previously described [24]. Equal amounts of proteins (25 μg) were separated on SDS-PAGE gels, transferred to polyvinylidene difluoride membranes (Millipore), and then incubated with a primary antibody (1:2,000) overnight at 4 °C and then incubated with an appropriate secondary antibody (1:2,000) at 25 °C for 1 h. The blots were detected with the Image Quant LAS 4000 mini system (GE Healthcare Bio-sciences AB, Inc., Sweden) after incubation with the ECL plus system (Amersham Biosciences, Sweden). Chemifluorescence was quantified with the use of the Quantity One software (Bio-Rad Laboratories). All results were normalized to GAPDH and expressed as relative values to those of the control group.

### Immunofluorescence assay

IPEC-1 cells treated with or without CA were fixed with 4% paraformaldehyde at 37 °C for 20 min, and then were incubated with a specific primary antibody against claudin-1, claudin-3, claudin-4, ZO-1, ZO-2 and ZO-3 for 16 h at 4 °C. Cells were washed three times with PBS, and then were incubated with an appropriate secondary antibody (1:100) for 1 h at 25 °C. Nuclei were stained by using Hoechst 33258 (1 μg/mL) for 10 min at 25 °C. The distribution of TJ proteins was visualized under a fluorescence microscope (Axio Vert. A1, Zeiss, Germany).

### Statistical analysis

Values are expressed as mean ± SEM. Data was analyzed by one-way ANOVA and the Student-Newman-Keuls multiple comparisons test, using the SPSS statistical software (SPSS for Windows, version 17.0). *P* ≤0.05 were taken to indicate statistical significance.

## Results

### Effects of CA on barrier function in the IPEC-1 cell monolayer

**Fig. 1:**
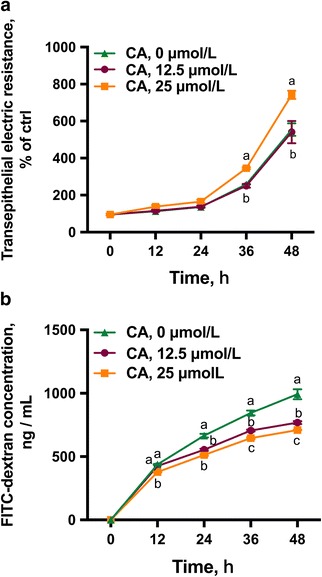
Effects of CA on intestinal barrier function in IPEC-1 cells. Cells were cultured for 24 h in the absence or presence of 12.5- or 25 μmol/L CA. **a** TEER and **b** paracellular permeability were then determined. Values are expressed as means ± SEM, *n* = 6. Means at a time point without a common letter differ, *P* < 0.05. CA, Cinnamicaldehye; IPEC-1, intestinal porcine epithelial cell line 1; TEER, trans-epithelial electrical resistance

As shown, incubation of cells with 25 μmol/L CA led to greater (*P* < 0.05) TEER at 36-48 h (Fig. 1a) when compared with controls. In contrast, no difference was observed between the cells treated with 12.5 μmol/L CA and the control cells at 12-48 h. Consistent with increased TEER, cells incubated with 25 μmol/L CA had reduced (*P* < 0.05) paracellular permeability, as indicated by FITC-dextran flux at 12-48 h (Fig. 1b) when compared with controls. Cells treated with 12.5 μmol/L CA had lowered permeability (*P* < 0.05), compared with the control cells at 24-48 h. Although both 12.5 and 25 μmol/L CA treatment led to decreased permeability in enterocytes compared with the controls, cells treated with 25 μmol/L CA had lower permeability (*P* < 0.05) when compared with cells incubated with 12.5 μmol/L CA at 36-48 h.

### Effects of CA on expression of TJ proteins in IPEC-1 cells

**Fig. 2:**
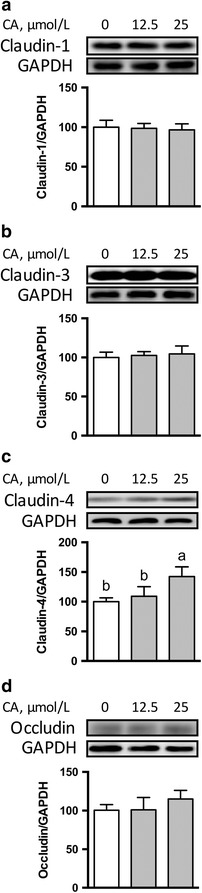
Protein abundances for claudin-1 (**a**), claudin-3 (**b**), claudin-4 (**c**), and occludin (**d**) in IPEC-1 cells. IPEC-1 cells were cultured in the absence or presence of 12.5 or 25 μmol/L CA for 24 h. Cells were collected and protein abundances were analyzed. Values are expressed as means ± SEM, *n* = 3. Means without a common letter differ, *P* < 0.05. CA, Cinnamicaldehyde; IPEC-1, intestinal porcine epithelial cell line 1

**Fig. 3:**
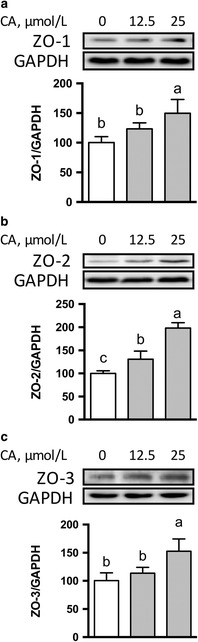
Protein abundances for ZO-1 (**a**), ZO-2 (**b**), and ZO-3 (**c**) in IPEC-1 cells. Cells were cultured in the absence or presence of 12.5 or 25 μmol/L CA for 24 h. Cells were collected and protein abundances were analyzed. Values are expressed as means ± SEM, *n* = 3. Means without a common letter differ, *P* < 0.05. CA, Cinnamicaldehye; IPEC-1, intestinal porcine epithelial cell line 1; ZO, zonula occludens

Compared with control cells, 25 μmol/L CA enhanced (*P* < 0.05) the abundance of proteins for claudin-4 (Fig. 2c) and ZO family proteins including ZO-1, ZO-2, and ZO-3 (Fig. 3). The protein abundance for ZO-2 (Fig. 3b), instead of other proteins, was enhanced by 12.5 μmol/L CA (*P* < 0.05) compared with that of the control. The protein abundances for claudin-1(Fig. 2a), claudin-3 (Fig. 2b), and occludin (Fig. 2d) were not affected (*P* > 0.05) by 12.5 or 25 μmol/L CA treatment.

### Effects of CA on the intracellular distribution of TJ proteins in IPEC-1 cells

**Fig. 4:**
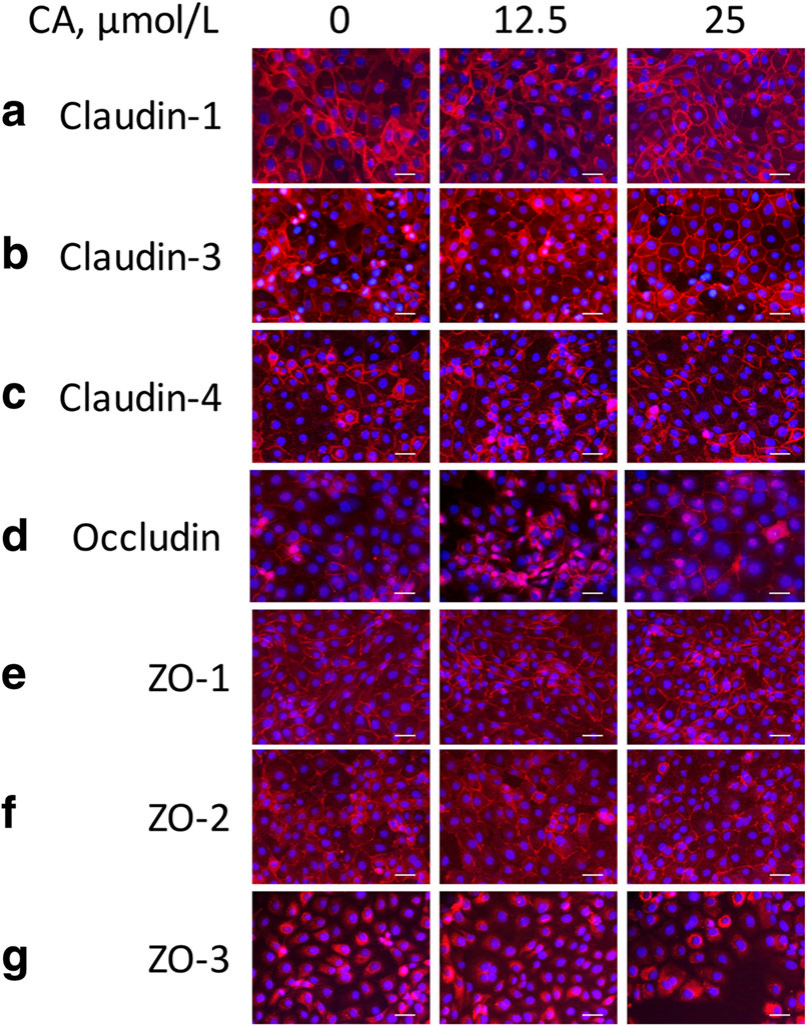
The distributions of TJ proteins claudin-1 (**a**), claudin-3 (**b**), claudin-4 (**c**), occludin (**d**), ZO-1 (**e**), ZO-2 (**f**), and ZO-3 (**g**) in IPEC-1 cells. Cells were treated as in Fig. 3, and immunofluorescence staining was performed to identify the distributions of the proteins. CA, Cinnamicaldehyde; IPEC-1, intestinal epithelial porcine cell line 1. Scale bar, 50 μm

The cellular distributions of TJ proteins were assessed by an immunofluorescence microscope. Treatment with 25 μmol/L CA promoted the localization of claudin-1 and claudin-3 (Fig. 4a and b) to the plasma membrane without affecting the localization of other TJ proteins, including claudin-4, occludin, ZO-1, ZO-2, and ZO-3, compared to the control cells (Fig. 4). In contrast, 12.5 μmol/L CA had no effect on the localization of TJ proteins determined in our study, such as claudin-1, claudin-3, claudin-4, occludin, ZO-1, ZO-2, and ZO-3 (Fig. 4). It should be noted that most of the ZO-3 was located at the nucleus membrane which was not affected by CA exposure (Fig. 4g).

### Effects of CA on the protein abundance for amino acid transporters

**Fig. 5:**
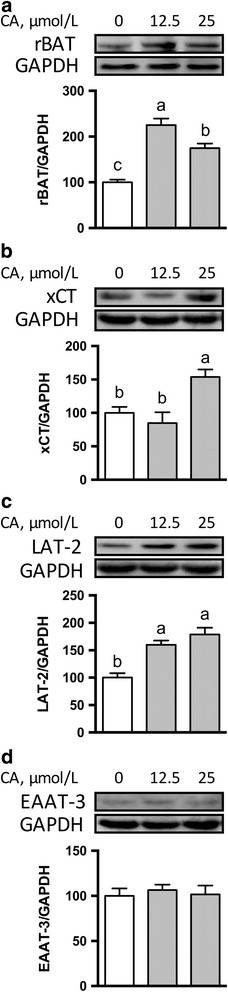
Protein abundances for rBAT (**a**), xCT (**b**), LAT2 (**c**), and EAAT3 (**d**) in IPEC-1 cells. Cells were cultured in the absence or presence of 12.5 or 25 μmol/L CA for 24 h. Cells were collected and protein abundances for amino acid transporters were analyzed. Values are expressed as means ± SEM, *n* = 3. Means without a common letter differ, *P* < 0.05. CA, Cinnamicaldehyde; IPEC-1, intestinal porcine epithelial cell line 1; ZO, zonula occludens

The active transport of amino acids is the major mechanism for their uptake into enterocytes [26–28]. We determined the protein abundance for the following amino acid transporters, EAAT3 (high-affinity glutamate transporter), LAT2 (arginine and leucine transporter), rBAT (basic amino acid transporter)*,* and xCT (acidic amino acid transporter) in IPEC-1 cells by Western blot analysis. The protein abundance for rBAT (Fig. 5a) and LAT2 (Fig. 5c) in IPEC-1 cells were enhanced (*P* < 0.05) by both 12.5- and 25 μmol/L CA, compared with the control cells. In contrast, 25 μmol/L CA increased (*P* < 0.05) the protein abundance for xCT in the intestinal epithelial cells, but 12.5 μmol/L CA had no effect (Fig. 5b). The protein abundance for EAAT3 was not affected (*P >* 0.05) by either 12.5 or 25 μmol/L CA (Fig. 5d), compared with the control cells.

## Discussion

In the present study, we have shown that CA, a key flavor compound in cinnamon essential oil, promoted the intestinal mucosal-barrier function as indicated  by increased TEER and decreased paracellular permeability. Western blot analysis revealed that cells treated with CA had enhanced protein abundances for TJ proteins, such as claudin-4 and scaffolding proteins. Moreover, the protein abundance for amino acid transporters, including rBAT, LAT2, and xCT, which are required for amino acid transport and absorption in enterocytes, were also enhanced by CA treatment.

CA, a natural compound isolated from the stem bark of *Cinnamomum cassia*, is widely used in food processing and animal diets due to its antioxidant, anti-microbial, and anti-diabetic attributes [2–4]. Studies in pigs, a widely used animal model for various disorders in humans, have demonstrated that CA supplementation enhances nutrient digestibility in pigs [10]. It remains largely unknown whether CA supplementation can have any effect on intestinal barrier integrity, thereby improving nutrient transport, absorption, and intracellular homeostasis.

To test this hypothesis, we first measured TEER, an indicator of intestinal epithelial integrity and permeability of intestinal epithelium. Incubation of the enterocyte with CA led to increased TEER and decreased FITC-dextran flux in intestinal porcine monolayers, suggesting a beneficial effect of CA on barrier function. Epithelial barrier function and paracellular permeability are primarily determined by epithelial TJ proteins [29, 30]. Transmembrane proteins (e.g., the claudin family protein, occludin) and peripheral membrane proteins (e.g., ZO-1, ZO-2 and ZO-3) have been identified as critical components of TJ proteins [31, 32]. Disruption of epithelial TJ proteins has been reported to be associated with multiple intestinal disorders [29, 33]. Consistently, restoration of TJ proteins by nutrients or prebiotics can improve mucosal barrier integrity and function in humans and animals [34]. We have recently found that dietary supplementation of glutamine prevented weanling stress-induced intestinal-mucosal barrier breakdown by augmenting TJ protein abundance [35], suggesting a functional role for amino acids in regulating mucosal barrier function.

In the present study, we found that CA regulates the protein abundance and cellular distributions of TJ proteins in intestinal cells. Specifically, the presence of 25 μmol/L CA led to enhanced protein abundances for claudin-4, ZO-1, ZO-2, and ZO-3, which are correlated well with augmented TEER values in IPEC-1 cells (Fig. 1). The claudin family proteins and ZO family proteins, play a crucial role in establishing  cell–cell contacts and maintaining paracellular permeability [36, 37]. Recent studies have demonstrated that the reduction of claudin family proteins is strongly associated with intestinal barrier disruption in rodents [38, 39]. The regulatory effects of CA on the protein abundance of TJ suggest that supplementation with CA might be a preventive strategy to maintain the appropriate function of the intestinal-mucosal  barrier. Another novel finding of our study is that CA treatment led to the distributions of claudin-1 and claudin-3 to the cellular plasma membrane (Fig. 4a and b **)** without affecting their protein abundances (Fig. 2a and b). Thus, CA regulates both the abundance and localization of TJ proteins in enterocytes. Considering that the disruption of TJ is caused by various stresses and pathogens in pigs [40], supplementation of CA may provide an effective nutritional strategy to alleviate mucosal barrier dysfunction. At present, the underlying mechanisms responsible for this effect remain incompletely understood [31]. More research involving our IPEC-1 cell model is required to answer this question.

In addition to providing physical and functional barriers to prevent the entry of bacteria, endotoxins, and other harmful substances from entering the blood circulation, appropriate amounts of TJ proteins maintains the integrity of the intestinal epithelium and, therefore, are  also required for the absorption of nutrients [15]. Amino acids released from the hydrolysis of dietary proteins and peptides in the lumen of the small intestine are transported across cell membranes by a complex system of multiple amino acid transporters [26–28]. A number of transporters have been identified on the apical surface of the mammalian small intestine that are responsible for the intestinal absorption of amino acids [26, 41, 42]. The defective intestinal uptake of amino acids leads to alterations in plasma amino acids, growth retardation, and the Hartnup disorder [26]. We have found that CA increased protein abundances for amino acid transporters, including LAT2, rBAT*,* and xCT in porcine enterocytes. This is the first study showing that this flavor compound has the ability to up-regulate the expression of amino acid transporters in enterocytes. The enhanced protein abundance for amino acids transporters might promote the transport and absorption of amino acids, which, in turn, stimulates  protein synthesis and contributes to the growth performance of pigs observed in previous studies [43, 44].

## Conclusions

In summary, studies with porcine enterocytes have revealed that CA improved the intestinal epithelial barrier integrity, as indicated by increased TEER and decreased paracellular permeability. This beneficial effect of CA is accompanied by enhanced distribution of specific TJ proteins in intestinal epithelial cells. Importantly, the protein abundance for amino acid transporters was enhanced by CA. Further studies with animal model are needed to validate this beneficial effect of CA on intestinal barrier function observed in the present study. Supplementation with CA might be a potential nutritional strategy to improve the intestinal mucosal barrier function and nutrient absorption in neonatal piglets.

## Abbreviations

CA: Cinnamic aldehyde
DMEM-F12: Dulbecco’s modified Eagle’s F12 Ham medium
EGF: Epidermal growth factor
FBS: Fetal bovine serum
HRP: Horseradish peroxidase
ITS: Insulin-transferrin-selenium
IPEC-1: Intestinal porcine epithelial cells-1
TEER: Trans-epithelial electrical resistance
TJ: Tight junction
ZO: Zonula occludens
